# Ground Waste Tire Rubber as a Total Replacement of Natural Aggregates in Concrete Mixes: Application for Lightweight Paving Blocks

**DOI:** 10.3390/ma14247493

**Published:** 2021-12-07

**Authors:** Matteo Sambucci, Marco Valente

**Affiliations:** Department of Chemical Engineering, Materials, Environment, Sapienza University of Rome-INSTM Reference Laboratory for Engineering of Surface Treatments, 00184 Rome, Italy; marco.valente@uniroma1.it

**Keywords:** ground waste tire rubber, rubber-cement materials, concrete paving blocks, physical properties, microstructure, mechanical testing, acoustic characterization

## Abstract

The use of waste materials as alternative aggregates in cementitious mixtures is one of the most investigated practices to enhance eco-sustainability in the civil and construction sectors. For specific applications, these secondary raw materials can ensure adequate technological performance, minimizing the exploitation of natural resources and encouraging the circular disposal of industrial or municipal waste. Aiming to design and develop lightweight paving blocks for pedestrian or very light-traffic purposes (parking area, garage, sidewalk, or sports surfaces), this paper presents the material characterization of rubberized cement mortars using ground waste tire rubber (0–1 mm rubber powder and 1–3 mm rubber granules) to totally replace the mineral aggregates. Considering recommended requirements for concrete paving members in terms of mechanical strength, water drainage performance, acoustic attenuation, and dynamic and energy absorption behavior, a comprehensive laboratory testing is proposed for five different formulations varying the sand-rubber replacement level and the proportion ratio between the two rubber fractions. Tests highlighted positive and promising results to convert laboratory samples into pre-cast members. The “hot” finding of the work was to prove the feasibility of obtaining totally rubberized mortars (0 *v*/*v*% of sand) with suitable engineering performance and enhanced eco-friendly features.

## 1. Introduction

The concept of “sustainability” is based on the implementation of strategies and practices in the industrial and economic fields aimed at hindering the depletion of natural resources and environmental pollution. As is well known, the civil engineering field involves activities such as the use of building materials from various natural raw resources, use of machinery and vehicles, demolition of existing structures, and land and forestry degradation which can impact the environment in many ways, including the generation of waste materials, hazardous emissions, noise pollution, and overconsumption of non-renewable virgin materials, including water and mineral aggregates [1]. In recent times, there is an increasing need for the design and development of mortar and concrete with an eco-friendly value. A simple and effective way to prevent the rapid depletion of natural source quarries (sand, gravel, and crushed stone) and maintain the ecological balance concerns the use of recycled materials, such as demolition wastes or industrial by-products, in place of natural aggregates. The environmental aspects are not the only reason for the use of alternative aggregates. Indeed, a lot of waste products, including waste glass powder [2], plastics [3], ground granulated blast furnace slag [4], wood ash [5], and crumb rubber from scrap tires [6] have properties suitable for being incorporated into cementitious matrices, bringing potential engineering functionalization in concrete materials, such as lightweight, higher durability, better shock absorption, and improved thermo-acoustic insulation properties.

Modern urbanization has contributed to an ever-increasing demand for the automotive sector, promoting a growing demand for tires, with global manufacturing being about 1500 million tires every year [7]. As a result, the world is projected to generate approximately one billion end-of-life tires each year [8]. Originally, many countries selected landfill disposal and incineration as the main methods for managing waste tires. However, these practices have proved highly ineffective in terms of economic impact, environmental consequences, and harmful effects on human health. Non-degradability, high flammability, and the release of fumes and toxic substances due to burning treatments are the main risk factors in the context of tire disposal [9,10]. Recently, several policies were established in Europe, based on the “clean” disposal, recycling, and the reuse of rubber from end-of-life tires [9]. For example, in Italy, the Legislative Decree n. 78 of 31 March 2020, defined “End of Waste” [11], officially recognizes recycled rubber as a secondary raw material and establishes a novel management system that enhances the use of granular rubber (obtained from grinding processes) in several fields, such as sports surfaces (flooring with improved elasticity and shock absorption properties), asphalt road pavements (high durable and anti-noise bitumen-based compounds), and building materials (lightness, sound-insulating, and vibro-absorbing components). The first promising results were obtained through the application of these “circular” waste management plans. According to the Ecopneus report (2019) [12], of the more than 350 million tons of end-of-life tires generated in Italy, 57% were sent for material recovery to manufacture ground rubber products intended for the above-mentioned application sectors, implying interesting benefits under the eco-sustainable point of view: 370 thousand tons of carbon dioxide (CO_2_) emissions were avoided, about 1.5 million m^3^ of water were saved, and up to 330 thousand tons of raw materials were not withdrawn. Generally, the application of organic wastes in basic materials used in the civil sector suffers by environmental concerns due to the heavy metals and polycyclic aromatic hydrocarbons (PAHs) contained in the waste organic components, limiting their recyclability and end-of-life management. Nowadays, regulations by Italian government regarding the second life of recycled aggregates contacting organic components are reported in the Ministerial Decree no. 203/2003, which defines a usage limit of 0.6% wt. in the case of polymer fractions.

Although granulated tire rubber has been repeatedly challenged in the past due to the suspected presence of some substances considered toxic and contained in components of vulcanized product, several studies [13,14] demonstrated tolerable ecotoxicity potentials (leaching of metals and PAH) with respect the limits imposed by the environmental regulations in force in Italy (Legislative Decree no. 152/2006). Among these, it is also worth mentioning a large-scale investigation conducted by Ecopneus in 2014 [15] aimed at evaluating the chemical and toxicological profiles of GTR deriving different European and non-European recycling plants. The analysis revealed a very limited content of PAHs in all the samples analyzed, recording concentration levels between 5 and 20 ppm, i.e., values from 100 to 10,000 times lower than the limit set by Registration, Evaluation, Authorization, and Restriction of Chemicals (REACH) regulations [16] for products destined to public sale. In addition, the study involved an in vivo exposure and risk assessment performed on artificial turf fields infilled with tire rubber granules, demonstrating that both during the installation of the infill (workers) and during sports practice (athletes), the incremental carcinogenic risk is significantly less (by an order of magnitude) than the recommended limits. Then, by using tire rubber in an embedded form as an alternative aggregate in concrete materials, the potential impact ecotoxicological impact would be even more minimized. The surrounding cementitious material can well confine the trace metals or volatile organics exited in rubber particles, avoiding their leaching. These aspects, combined with the new cleaning, pretreatment, and monitoring directives for recycled rubber aggregates required by “End of Waste” decree, could represent an attractive challenge to encourage the circularity/recyclability of the rubber-based materials and their integration in the construction sector.

The use of ground tire rubber (GTR) as a concrete aggregate replacement, termed rubber concrete (RC) technology, has been the subject of a considerable number of small-scale or laboratory studies over the past 30 years [6,17,18,19]. Common remarks were highlighted regarding the effect of GTR on the technological properties of cementitious materials: improved energy absorption capacity and ductility, greater thermo-acoustic insulation, higher sound absorption, improved freeze-thaw resistance, higher permeability, and drainage properties. Moreover, recently, innovative implementations of RC technology were proposed, including the development of 3D-printable rubber-cement compounds [20,21] and use in eco-friendly Geopolymer concretes [22]. However, the applicability of cement-based materials modified with recycled rubber is still not well consolidated and limited for non-structural civil or architectural proposals. The main drawback is related to the remarkable loss in mechanical strength, which depends on the weak rubber-cement bonding condition, the softness and lightweight properties of polymer aggregates compared to the mineral ones, and the rubber’s hydrophobic tendency to incorporate air, and therefore porosity, into the matrix. In this framework, the particle size gradation, shape, percentage replacement level, and compatibilizing pre-treatments on GTR, play a key role in the final characteristics of the material [17].

**Figure 1:**
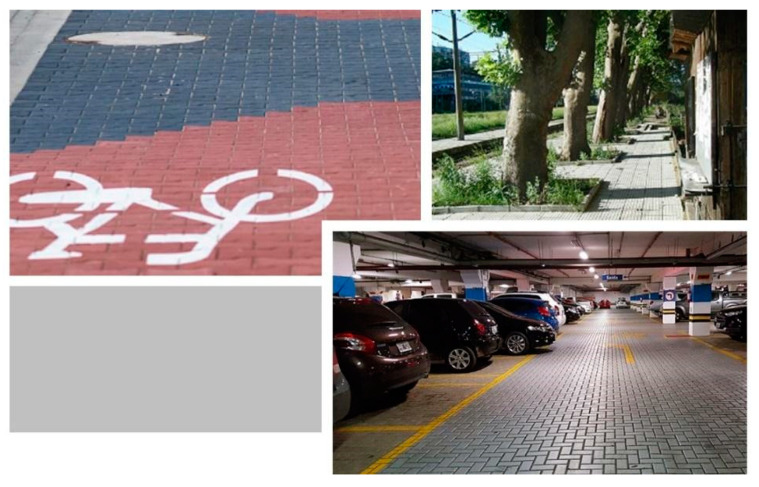
Paving blocks for pedestrian or very-light traffic applications.

Based on the findings and research results described above, the purpose of this work was the determination of feasibility using Portland-based rubberized formulations in the fabrication of lightweight paving blocks designed for pedestrian or very light-traffic applications, including indoor parking areas, garages, sidewalks, or surface for sports activities (Figure 1) as reported in UNI 11714-1:2018 technical standard [23], which classifies the pavement surfaces according to the types of transit and traffic load. These pre-cast components involve technological properties that potentially fit with the peculiarities of rubber-functionalized cementitious materials: low-strength requirements, permeability for drainage purposes, noise attenuation, and impact energy absorption.

The recent scientific literature provides some preliminary studies on the development and characterization of new eco-sustainable cementitious mixes, based on recycled aggregates, potentially suitable in the production of paving elements. Limantara et al. [24] developed cement-based mix designs by using coconut fibers and crushed shells as aggregates in different ratios, demonstrating compliance between strength and permeability test results with the Indonesian technical requirements for parking areas. Agyeman et al. [25] evaluated the potential of using grounded plastic waste (polyethylene bags, sachet water bags, and bubble packaging films) as a binding material to totally replace the cement, revealing physical and mechanical performances in agreement with recommended values for non-traffic applications (walkways, footpaths, and landscapes) in Ghana. Caetano et al. [26] used fine shredder scrap from end-of-life vehicles to produce concrete paving blocks, totally replacing the natural fine aggregates (sand), demonstrating their potential use for pedestrian traffic and light vehicles in accordance with Brazilian standards. Tataranni [27] proposed a complete laboratory characterization on paving blocks produced by the alkali-activation process of waste basalt powder. From the experimental results, it has been noted that the presence of a certain number of mineral fractions is essential to achieve strength and permeability requirements compliant with the European recommendations.

Up to now, a limited number of works have been published on the use of GTR to totally replace the natural fine and coarse aggregate in the manufacturing of concrete paving units. Innovatively, in this study, the development of “sand-free” mix designs was attempted to obtain rubber-cement formulations with improved eco-friendly features and suitable performance for the applications under investigation. Furthermore, almost all research on recycled concrete paving members do not cover some key technological features, including the dynamic mechanical response of the material, for example, resulting from the tire–pavement interaction during the vehicle motion or the pedestrian walk, the acoustic properties, for example, related to the attenuation of the noise generated during the car parking operation or from the urban soundscape, and the drainage performance, which are desirable to ensure the rainwater runoff and avoid excessive water accumulation on the pavement surface. For this purpose, a laboratory characterization was conducted on four different GTR-cement formulations, obtained by replacing the mineral part with two types of rubber fractions (0–1 mm rubber powder and 1–3 mm rubber granules). By investigating different rubber mix proportions and rubber-sand volumetric replacement levels (0% *v*/*v*, 50% *v*/*v*, and 100% *v*/*v*), the effect of size gradation and polymer content on the physical, mechanical, and acoustic properties of the material was evaluated and compared with acceptance requirements reported in recent literature [28,29,30], which specifies the recommended performance for concrete materials suitable in light-traffic, pervious, and anti-noise paving applications.

## 2. Materials and Methods

### 2.1. Materials and Specimens Preparation

**Figure 2:**
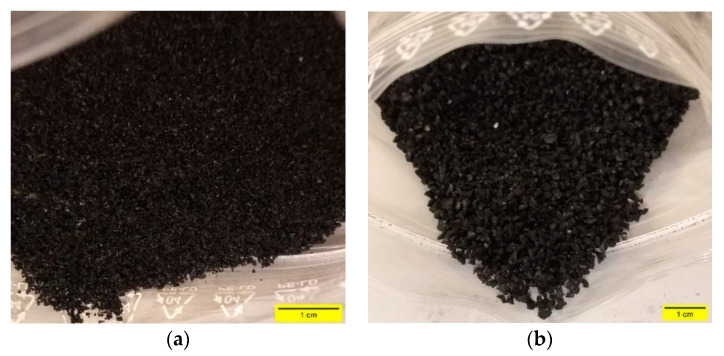
Photographs of (**a**) RP and (**b**) RG.

**Figure 3:**
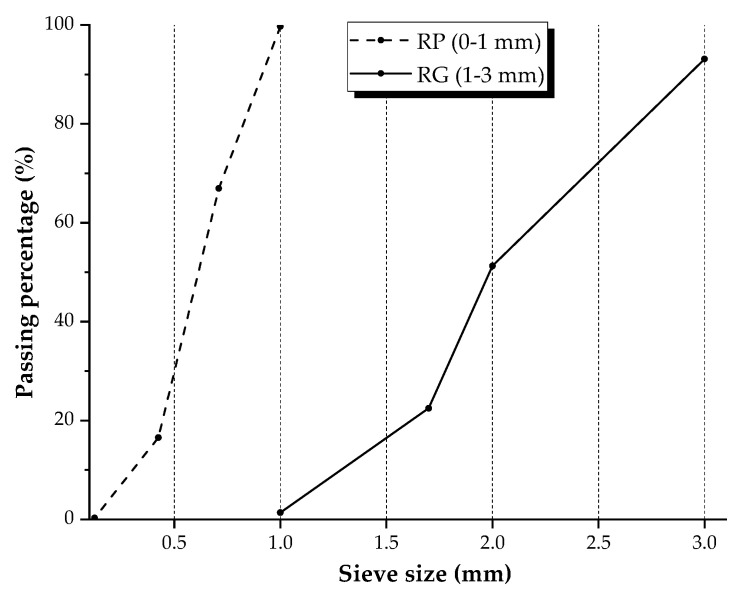
Particle size distribution of RP and RG.

The GTR fractions, obtained from the mechanical shredding of end-of-life tires, was supplied by European Tyre Recycling Association (ETRA, Brussels, Belgium), a pan-European body that represents the independent tire recycling sector in Europe involving the major granular tire rubber production plants operating on the territory. Two particle size fractions were studied: 0–1 mm rubber powder (RP) and 1–3 mm rubber granules (RG). Figure 2 shows the photographs of used rubber aggregates. Sieve analysis was performed using different mesh sizes following the technical indications for sampling and sieving time reported in DIN 51701 [31] and ARIZ 714c [32] standards, respectively. The grading curves are plotted in Figure 3. The density of GTR was evaluated by pycnometer method by employing ethylic alcohol as the fluid of know density to prevent floating of polymer particles. An average value of 1144 kg/m^3^ was measured. The water absorption, calculated as the ratio of the difference between the weight of the rubber aggregates in saturated surface dry (SSD) condition and oven-dry condition (110 °C for 24 h) to the weight of oven dry aggregates, was 9.6%. The same testing methods were used to evaluate the density and water absorption of mineral aggregates (see below).

The base cementitious formulation (CTR) consisted of Type I Portland cement (strength grade of 42.5 R in accordance with UNI EN 197-1 standard [33] and density of 3280 kg/m^3^), 0/0.4 mm fine limestone sand (density of 2476 kg/m^3^ and water absorption rate of 19.9%), tap water, and a specific proportion of chemical admixtures, including Silica fume-based thixotropic additive (SF), Polycarboxylate ether-based superplasticizer (PES), Aliphatic-based water reducing agent (WRA), and Calcium oxide-based expansive agents (CEA), whose amount was kept constant in all formulations. Starting from the reference mix, four rubberized formulations were designed by volumetrically replacing the sand with the two polymer fractions in different ratios. The mixtures constituents used for the sample preparation are provided in Table 1.

**Figure 4:**
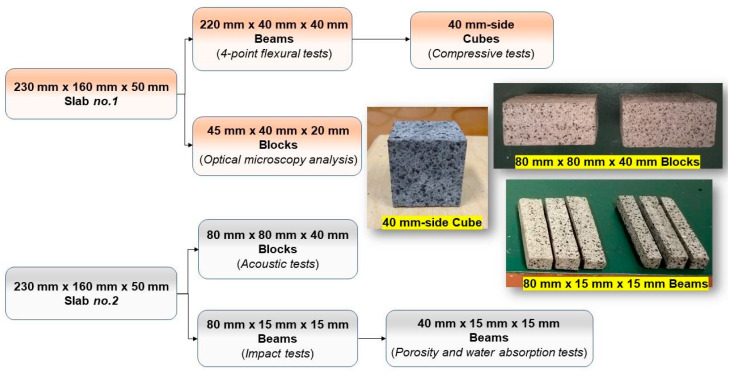
Set of specimens for experimental testing.

As reported in Table 1 above, a certain amount of RP, having a size gradation close to the natural aggregate, was always preserved in all GTR-cement mixes. As known, the fine fraction in the concrete mix designs is crucial to enhance the compactness of the material, which affects its load-carrying capacity [34], and to ensure a better cohesion of the paste, increasing the aggregate packing density and the static stability of the cementitious compound [35]. The effect of coarser rubber aggregates (RG) was investigated in RP50-RG50 and RP25-RG75 formulations, where the RP:RG proportion ratios were 1:1 and 1:3, respectively. For each mortar mixture, two samples were cast in prismatic molds to obtain slabs with a length of 230 mm, a width of 160 mm, and thickness of 50 mm. The adoption of “non-standard” molds but dimensionally like pre-cast blocks has allowed a qualitative evaluation of the fresh properties of the mixes, adjusting, and fixing the free water content to obtain suitable fluidity for the potential casting operations in paving block formworks. After 24 h, all the samples were removed from the molds and subjected to curing treatment in clean water at room temperature for 28 days. The set of specimens for the experimental tests (see the flowchart in Figure 4) were extracted from the hardened slabs by wet cutting with a Labotom-3 abrasive cut-off saw (Struers Inc., Cleveland, OH, USA) equipped with a NX30 Al_2_O_3_ resinoid cutting disk (NX-MET, Echirolles, France). Similar procedure of specimens’ extraction from concrete blocks was carried out by Elfordy et al. [36].

### 2.2. Experimental Testing Program

#### 2.2.1. Permeable Porosity, Water Absorption, and Pore Size Distribution

**Figure 5:**
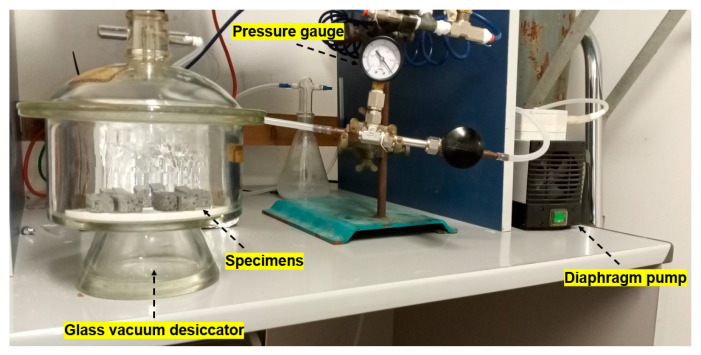
Testing system for vacuum saturation method.

Vacuum saturation method (ASTM C1202 standard [37]) was employed to assess the permeable porosity (*Φ*) and water absorption (*WA*) of the samples under investigation. The test apparatus is illustrated in Figure 5. After drying in an air oven at 105 °C for 24 h, the specimens were weighted (*M_d_*) and placed in a glass vacuum desiccator connected to a Divac 0.6 L diaphragm pump (Leybold, Cologne, Germany). By pumping, the container was evacuated for 30 min, reaching a vacuum level of 40 mbar. Then, the desiccator was backfilled with tap water to cover the specimens, which were left to soak for a further 30 min. After returning the system to atmospheric pressure conditions, the samples were kept immersed for 3 h. An ME54 analytical balance (Mettler Toledo, Columbus, OH, USA) was equipped with a kit for weight measurements by hydrostatic method, to evaluate the mass of the specimens immersed in water (*M_w_*) and the mass of the saturated specimens in air (*M_s_*).

For each formulation, four beams were tested and the average values of *Φ* and *WA* were computed according to the following Equations (1) and (2):

(1) $$\Phi =\frac{M_{s}-M_{d}}{M_{s}-M_{w}}\times 100$$

(2) $$WA=\frac{M_{s}-M_{d}}{M_{d}}\times 100$$

**Figure 6:**
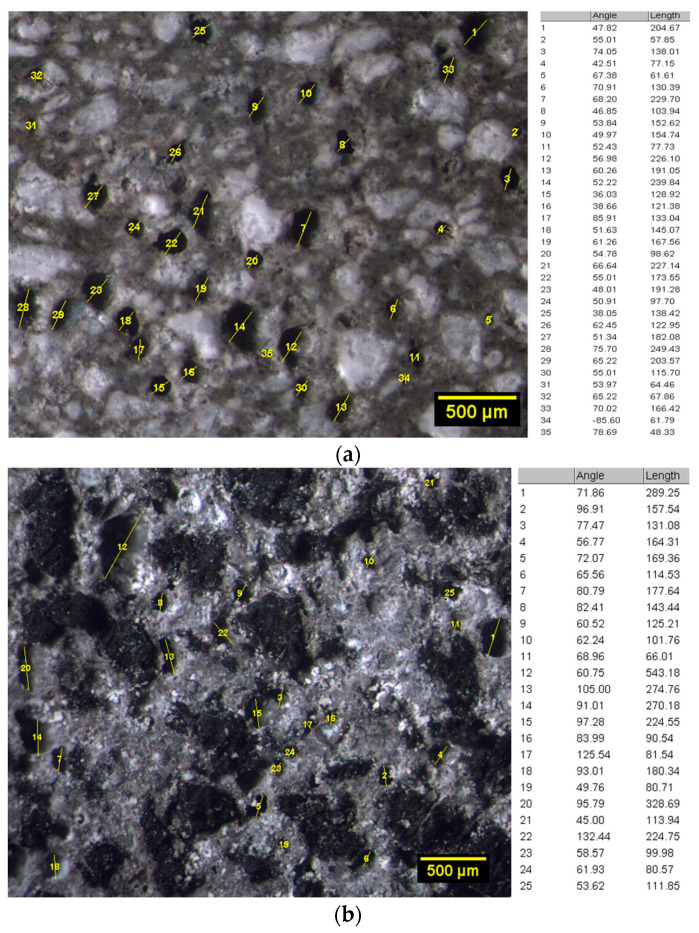
Optical microscopy image processing for pore size measurements: (**a**) CTR and (**b**) RP100 samples.

A quantitative analysis of pore size distribution was conducted by optical microscopic investigation. Test specimens were examined by a MS5 stereomicroscope (Leica, Wetzlar, Germany) and the digital processing of the micrographs was conducted by ImageJ software (NIH, Bethesda, MD, USA). For each mortar formulation, four cross-section images (16× magnification) were acquired, and the pore size distribution was computed by measuring the effective diameter of each void. The pore cumulative frequency (based on the number of air pores detected) was calculated in the macro-porosity range from 100 μm to 300 μm. Examples of optical micrographs processing for the pore size evaluation were reported in Figure 6a,b, respectively for REF and RP100 samples.

#### 2.2.2. Scanning Electron Microscopy

Scanning electron microscopy (SEM) analysis was conducted using a Mira3 FEG-SEM (Tescan, Brno, Czech Republic). Prior to the study, each sample was cut into small pieces of approximately 1 cm. Subsequently, the fragments were fixed, through a carbon adhesive tape, on a metallic stub and then made conductive by graphite sputtering (EM SCD005 vacuum sputter coater, Leica, Wetzlar, Germany).

#### 2.2.3. Static Mechanical Testing: Four-Point Flexural and Compressive Tests

Flexural test was conducted on three beams (220 mm × 40 mm × 40 mm) for each mix. The test was performed on a Z10 (10 kN load cell) universal testing machine (Zwick-Roell, Ulm, Germany) using a four-point configuration with 180 mm span length (a reference to ASTM C 348 [38]). A sensor arm (extensometer) was attached directly to the underside of the specimens for accurate measurement of the bending deflection. Uniaxial compressive test was conducted on 40 mm-side cube specimens (six samples for each formulation) extracted from broken beams in bending. According to ASTM C 109/109M standard method [39], the test was performed by using a Z150 (150 kN load cell) testing system (Zwick-Roell, Ulm, Germany). In both mechanical tests, the preload was 5 N, and the loading rate was 1 mm/min. The test parameter selection and the acquisition of the load–strain data were carried out by TestXpert computer-controlled testing software (Zwick-Roell, Ulm, Germany). The discussion of the mechanical results was combined with the average unit weight (*ρ*) values obtained as the mass-to-volume ratio.

#### 2.2.4. Dynamic Mechanical Testing: Charpy Impact Test

**Figure 7:**
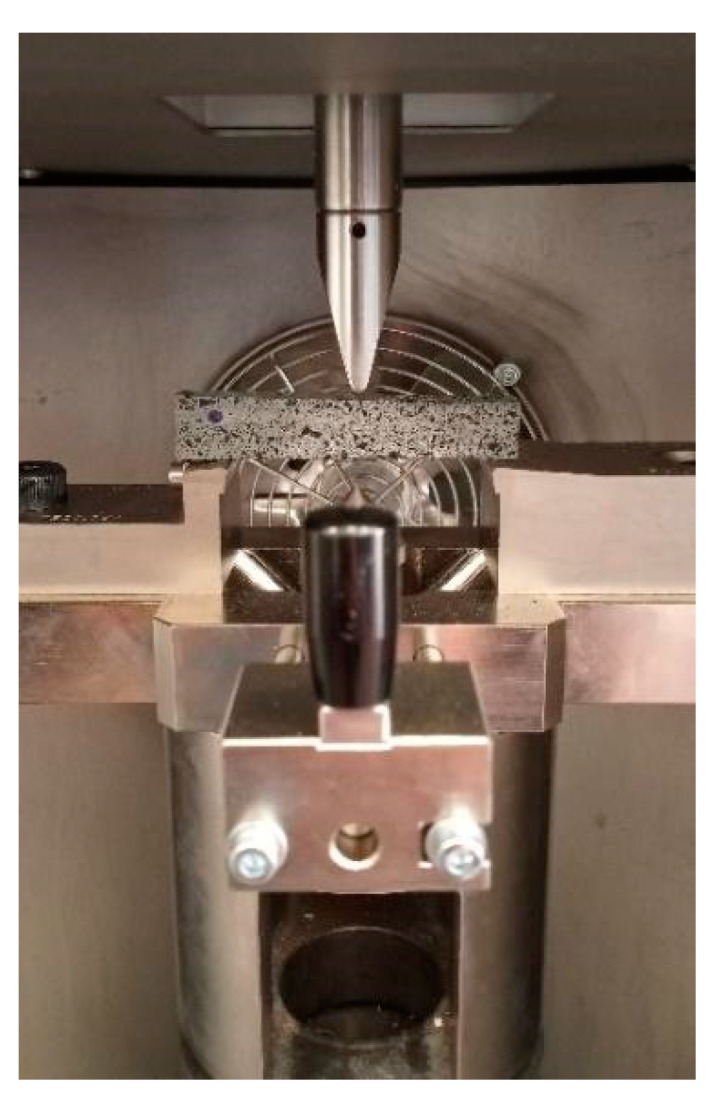
Experimental set-up for Charpy impact test.

Beam specimens (80 mm × 15 mm × 15 mm) were subjected to an impact test in compliance with ISO179-2 standard method [40] in an edgewise condition. An instrumented drop-tower impact machine (Instron/CEAST 9340, Pianezza, Italy), equipped with a Charpy striker’s head impactor, was used for this purpose (Figure 7). Three samples for each mix type were impacted at the kinetic energy of 13.5 J and impact speed of 2.9 m/s, considering a span length of 62 mm. The force–displacement curves and the dynamic parameters of the material in terms of absorbed energy were recorded by the DAS64K acquisition system. Impact energy absorbed at break (*E_b_*) was calculated as the area under the impact force–displacement curve from the rise point of the impact load to the first occurrence of zero load after the maximum peak.

#### 2.2.5. Acoustic Characterization

To investigate the influence of GTR addition on the acoustic behavior of the mortars, a custom-made impedance tube was designed and constructed in accordance with the technical indication reported by da Silva et al. [41]. The measurement system was composed of the following parts: (a) two complementary Poly (vinyl chloride) (PVC) tubes to form a 190 cm-long duct with an inner diameter of 16 cm; (b) a Polyethylene (PET) sound-insulating sheath (Fortlan-Dibi, Reggio Emilia, Italy) placed to cover the tube in order to improve the vibro-acoustic insulation of the system during the test; (c) a trapezoid-shape wooden acoustic box filled with Polyurethane (PU) acoustic foam (Paulstra-Hutchinson, Levallois-Perret, France) and located at one end of the duct; (d) a metal frame as a sample holder; (e) a rigid termination; and (f) a series of holes drilled on the tube acting as microphone locations.

A 30W MPA30BT loudspeaker (Behringer, Willich, Germany) was contained in the acoustic box and acted as an acoustic source. The geometry of the box and the presence of the sound-absorbing foam allowed it to minimize internal reflections and resonances, increasing the damping factor inside. The transduction of the sound signals was conducted by ECM800 ¼” condenser microphones (Behringer, Willich, Germany). A Scarlett 2i4 audio interface (Focusrite, High Wycombe, UK) was used for the processing of audio signals during the experiments. Room EQ Wizard software (GIK Acoustic, Atlanta, GA, USA) was employed for data analysis.

**Figure 8:**
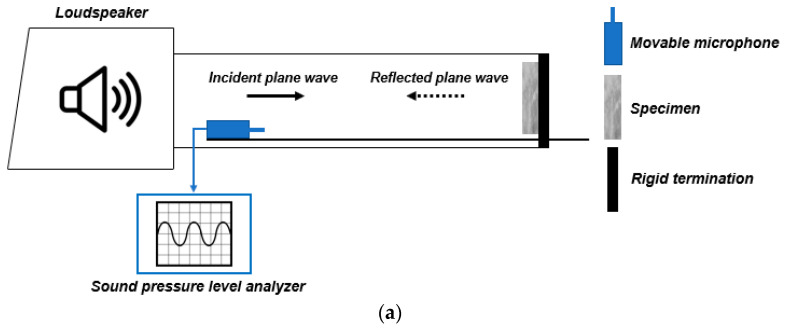

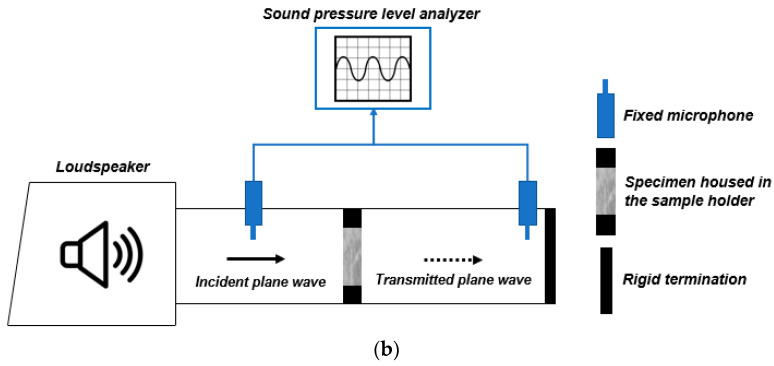
Schemes of the impedance tube configuration for (**a**) acoustic absorption measurements and (**b**) acoustic flow resistivity tests.

Depending on the type of test, specific experimental configurations were considered. Acoustic absorption measurements were performed by the standing wave ratio method (ASTM C384-95 standard [42]), according to the measurement scheme illustrated in Figure 8a. An incident sine wave acoustic signal was generated by the loudspeaker placed at one end of the tube. The other end-side was terminated with the test specimen backed with the reflective termination. A single microphone was anchored to a metal rod and manually moved along the duct recording the maximum and minimum sound pressure of the standing wave generated by the combination of incident and reflected waves. The diameter (*D*) and length (*L*) of the tube governed the operating frequency range (*f_min_* < *f* < *f_max_*) from the following Equations (3) and (4):

(3) $$f_{max}=\frac{0.586\times c}{D}\;$$

(4) $$f_{min}=\frac{0.750\times c}{L-D}\;$$

where *c* is the sound velocity (347.12 m/s) at the laboratory temperature of 27 °C. For the tube used in this study, *f_min_* and *f_max_* were 150 Hz and 1270 Hz, respectively. The normal incidence absorption coefficient (*α*), in third-octave bands, from 250 to 1250 Hz was determined by Equation (5):

(5) $$\alpha =\frac{4\times 10{\;}^{\frac{\Delta L}{20}}}{{\left(10{\;}^{\frac{\Delta L}{20}}+1\right)}^{2}}$$

where ∆*L* is the difference between the maximum and minimum sound pressure level measured along the tube for each frequency investigated. An additional parameter, which provides a further evaluation of the acoustic absorption ability for engineering purposes, is the noise reduction coefficient (NRC) [43]. NRC was calculated as the arithmetic average of *α*-values determined at the central frequencies (e.g., 250 Hz…1250 Hz).

Acoustic flow resistivity (*σ_a_*) is a physical quantity that characterizes the acoustic absorption and sound propagation of an absorbing medium and is a key factor in modeling the acoustic performance of the poro-acoustic materials [44]. In this work, *σ_a_* was computed in accordance with the method proposed by Ingard and Dear [45]. The experimental set-up (Figure 8b) involved the testing material housed in the sample holder and located in the middle of the tube. Two microphones were fixed upstream and downstream of the specimen: the first one in the microphone position closest to the loudspeaker (microphone-acoustic box distance of 75 cm), while the other one near the rigid termination (microphone-acoustic box distance of 155 cm). Using a low-frequency (<100 Hz) Log swept sine acoustic signal, *σ_a_* was determined by measuring the sound pressure loss across the specimen as follows (6):

(6) $$\sigma_{a}=\frac{\rho_{0}\times c}{t}\times 10^{\frac{\left(L_{i}-L_{t}\right)}{20}}$$

where *ρ*_0_ = air density at laboratory temperature of 27 °C (1.18 kg/m^3^), *t* = thickness of the specimen (m^2^), *L_i_* = sound pressure level in front of the specimen (dB), and *L_t_* = sound pressure level behind the specimen (dB). For each mix, *σ_a_*-values of two samples were measured and averaged.

#### 2.2.6. Statistical Analysis by One-Way ANOVA Test

The analysis of variance (ANOVA) is performed to verify the effect, in terms of statistical significance, of independent variables on dependent ones and whether significant interaction effects exist among independent variables in a set of experimental data. If only one influencing factor is involved in the study, the analysis refers to one-way ANOVA test. In this work, OriginPro software (OriginLab, Northampton, MA, USA) was used to investigate whether the effect of GTR size gradation (influencing factor or independent variable) significantly affects the physical–mechanical properties of rubberized mixtures (response factors or dependent variables). Specifically, the three formulations with total sand-rubber replacement were involved in the study (RP100, RP50-RG50, and RP25-RG75), in which the level of coarser polymer fraction (RG) was gradually varied (0% *v*/*v*, 50% *v*/*v*, and 75% *v*/*v*, respectively) in relation to the finest one (RP). Conventionally, the significance level (*p*-value) was kept at 0.05 for the statistical evaluation of the experimental results, representing 95% level of confidence. A *p*-value less than 0.05 indicates the statistically significative effect of RG addition on material performances.

## 3. Results

### 3.1. Permeable Porosity, Water Absorption, and Pore Size Distribution

In the discussion of *Φ* and *WA* experimental results, the following competitive factors must be considered:

- Water-to-cement (w/c) ratio. GTR-cement formulations showed mixing water amounts gradually lower than reference sample. This evidence is attributable to the smoother and more regular surface texture of rubber particles than sand and their non-absorbent nature [46]. Reduced w/c ratio determines a lesser capillary porosity in the hardened material [47].
- Nature of rubber aggregates. Hydrophobic characteristics of polymer aggregates promote the affinity to repel water and trap air onto its surface. Entrapped air, usually made up of irregular pores, increases the *Φ* percentage into the rubberized mortars [48].
- Rubber-cement adhesion. As previously mentioned, the bond between rubber particles and cement paste is poor. As a result, the generation of line-like pores in the interfacial transition zone (ITZ) is promoted. In addition, these interfacial defects encourage the initiation of permeable microcracks in the matrix due to the hygrometric shrinkage of the material during curing [48].
- Pore size reduction. The rheological properties of fresh cementitious materials are directly affected by the w/c ratio. The lower the water/cement ratio, the greater the mix viscosity. High viscosity impedes the coalescence of air bubbles into the paste, hindering the enlargement of the voids in the matrix [49]. For this reason, the progressive decrease in w/c ratio in the rubberized samples results in a refinement of the global macro-porosity detected by optical microscopy (OM) imaging analysis (Table 2). Based on this investigation, the pore sizes are mainly ranged between <100 μm and up to more than 300 μm. The influence of rubber on the reduction in the pore size is evident in the samples with total sand-polymer replacement, where the contribution of the finer porosity (<200 μm) is predominant.

**Figure 9:**
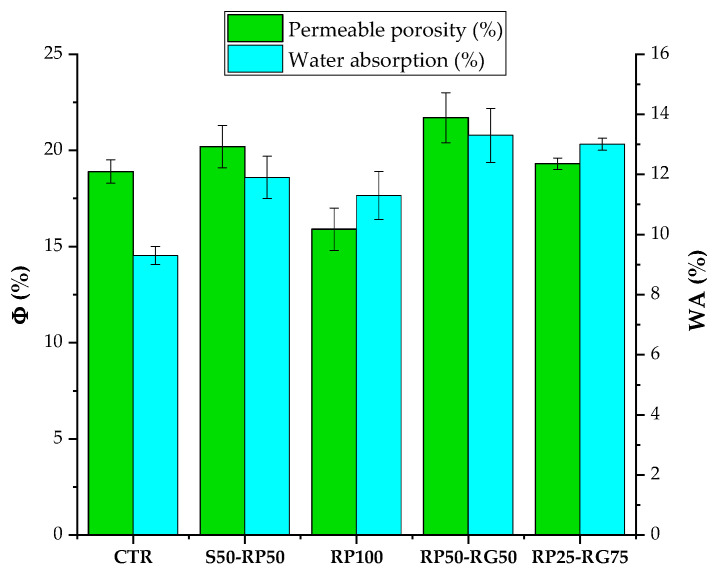
*Φ* and *WA*: Test results. Dispersion lines in the plot represent the standard deviation.

No significant variations in *Φ* and *WA* can be detected between CTR and rubber-cement formulations (Figure 9). This evidence can probably be attributed to a functional balance between incentivizing (air-entraining effect of rubber and ITZ voids) and contrasting (reduced w/c ratio and fine pore size gradation) factors on the porosity development and absorption properties. *Φ*-value slightly increases from 18.9%, in CTR, to 20.2%, 21.7%, and 19.3%, in S50-RP50, RP50-RG50, and RP25-RG75, respectively. The same growing trend can be found for *WA*. *WA*-value is 9.3% in CTR mix and reaches values of 11.9%, 13.3%, and 13.0% in S50-RP50, RP50-RG50, and RP25-RG75, respectively. Opposite behavior can be observed in RP100 mix, where the lowest *Φ*-value (15.9%) and, therefore smallest absorption rate (11.3%) among the rubberized samples, are recorded. In the first hypothesis, these results could be related to the better adhesion of the fine polymeric fraction with the cement matrix compared to coarse one, resulting in a lower impact of ITZ porosity. Furthermore, micro-filling ability of the small rubber particles can reduce the air void fraction of the matrix, making a more compact material [49].

Although porosity and water absorption are properties to be minimized in the case of load-bearing applications, both to ensure suitable mechanical strength and to increase the service life of the concrete elements (decrease the permeability of deteriorating agents and increase the corrosion resistance of steel-reinforced cement materials), their enhancement results in attractive requirements for the design of paving units in parking lots. As well reported by Seo et al. [28] and Grubeša et al. [29], pervious and permeable paving members are strongly functional in terms of water drainage against flooding or rainwater and resistance to freeze-thaw cycles, requiring a void ratio in the range of 15–30%.

### 3.2. SEM Analysis

**Figure 10:**
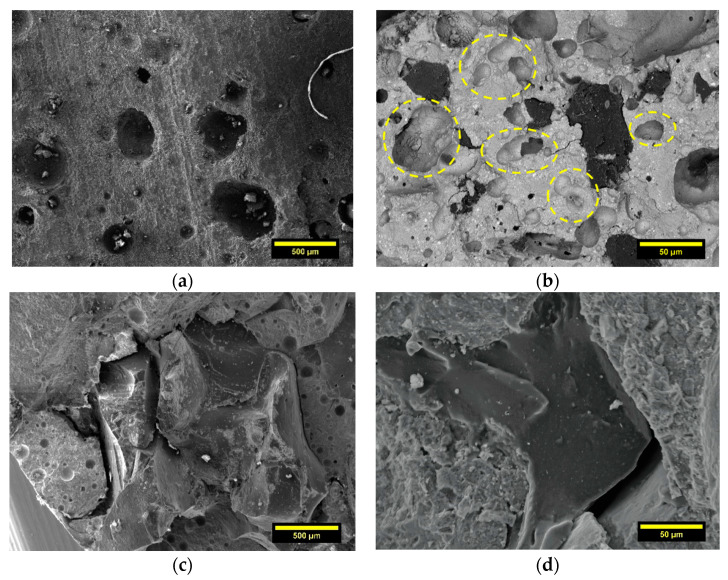
SEM analysis: (**a**) spheroidal pore system in CTR sample, (**b**) irregular macropores around the rubber particles in RP100 mix, (**c**) weak adhesion and porous ITZ between RG and cement paste, and (**d**) partial interfacial bonding between RP and cement matrix.

With the aim of supporting the experimental results of *Φ* and *WA*, SEM analyses were performed to evaluate the microstructural difference between CTR and rubberized samples and the variations in ITZ as a function of rubber size gradation. The secondary electron (SE) image, in Figure 10a, shows the typical spheroidal morphology of pore in CTR sample, which is associated with the air accidentally incorporated during the mixing of fresh material. Conversely, the backscattered electron (BSE) micrograph, in Figure 10b, highlights irregular voids in the matrix due to the addition of GTR. In agreement with Angelin et al. [50], irregular porous formation is intimately associated with the air trapped by the polymer aggregates. Figure 10c,d show the influence of rubber size gradation on the adhesion properties with the cement paste. The SE image, in Figure 10c, reports the poor interfacial cohesion between RG and matrix, resulting from the smoother surface texture and lower surface area of the coarser fraction than the fine one. A greater number of mechanical grinding cycles to obtain the finest fraction (RP) make the surface of the particles more rough and jagged. As shown in Figure 10d, this morphology slightly enhances the anchoring with the matrix, resulting in an overall more compact and continuous interface. However, where the particle exhibits a smoother micro-texture (see the low right corner in the image), this binding effect disappears, leading to interfacial gaps. The relationship between size gradation and rubber-cement adhesion was demonstrated by the authors in previous research [20,51]. In this regard, the use of supplementary cementitious materials (such as silica fume) or the application of physical and chemical pre-treatments (silane coupling agents, NaOH, or UV-based treatment) on rubber are widely explored practices to improve the interfacial GTR-cement bonding. However, further investigations are still required to find cost-effective, efficient, and non-complicating for concrete technology production methods of ITZ improvement, which are important concerning the field application of rubberized concrete [52].

### 3.3. Static Mechanical Properties: Compressive Test

The compressive strength (*σ_c_*) reduction in rubberized concrete or mortars is evidence unanimously shared by researchers operating in this field [6,10,17,18,19,20,21,22,46,47,51,52,53]. According to the reported literature, the reasons behind the negative effect of GTR on mechanical performance are summarized as follows: (a) low stiffness and higher deformability of the polymer particles than mineral aggregates, which results in high-stress concentrations and premature failure of material under load; (b) the weak bonding between rubber and cement paste, which acts as a structural defect; (c) the replacement of sand or other stone aggregates, which play a fundamental role in the strength of concrete materials, with “weaker” particles; (d) the increasing in air content into the cementitious compound, due to the water-repelling nature of rubber; and (e) the strong unit weight reduction resulting from the incorporation of lightweight aggregates in the cement matrix.

**Figure 11:**
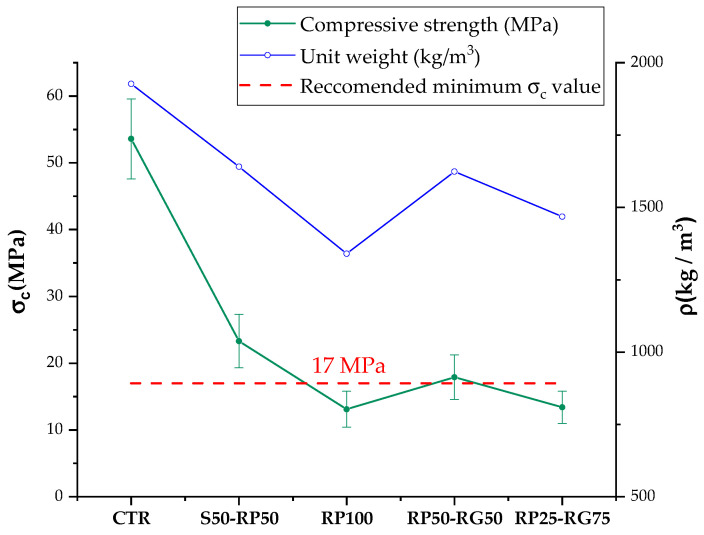
Compressive strength and unit weight trend: Test results.

In Figure 11, *σ_c_* results of CTR and rubberized samples are plotted in relation to the *ρ*-values. Maximum *σ_c_* recorded is for CTR mix with 53.60 MPa. By replacing sand with 50 *v*/*v*% of RP (S50-RP50 mix) results in a drop by 57%. As expected, the total sand-rubber replacement in RP100, RP50-RG50, and RP25-RG75 involves a more marked reduction in *σ_c_*-value (76%, 67%, and 75%, respectively), following the trend of *ρ*-values. Although from the results in Figure 9, RP100 mix provides a less porous microstructure, its greater reduction in unit weight is attributable to the higher overall content of rubber incorporated in the material, due to the higher packing degree (per unit of volume) in the cement paste of the finest fraction with respect RG. Two interesting observations can be drawn from these findings:

- Exploring the scientific literature, few contributions have attempted to completely replace the mineral aggregates with recycled rubber, due to the considerable mechanical losses found in material performance, severely limiting their application. Wu et al. [17] demonstrated that *σ_c_* decreased by 87% when chipped tire rubber (<20 mm) replaced the natural coarse aggregates with 100 *v*/*v*%. Atahan and Yucel [16] investigated the effect of replacing up to 100% of mineral aggregates with two gradations of crumb rubber: fine (1.3 mm maximum size) and coarse (13 mm maximum size) fractions. Rubberized concrete containing 100% rubber provides a strength reduction of almost 90% compared to the reference sample (0% rubber). Raffoul et al. [19] studied the performance of different cementitious mixes, incorporating fine (0–5 mm) and coarse (5–20 mm) rubber in various replacement ratios. The use of the fine polymer fraction in total replacement of the aggregate led to a reduction of about 85%, while the coarse fraction revealed a strength loss of 86%. From the comparison with the results mentioned above, the rubber-cement mixes investigated in this research appear to be more performing in terms of mechanical behavior, probably by virtue of the appropriate selection of rubber size gradation used to replace the natural aggregates. In this regard, RP (0–1 mm range size), with its sand-like granulometry and high specific surface, preserves the compound rheology and promotes the microstructural compaction of the hardened material (as shown in Figure 10d). RG (1–3 mm range size) prevents excessive *ρ* reduction (by considering the same rubber-sand replacement level) and, as a coarse fraction, has a better influence on the strain capacity of rubberized mortars [21]. To reach “sand-free” cementitious mixes with adequate mechanical performance results in beneficial implications from an environmental point of view, in terms of tire recycling, valorization, and savings of natural resources.
- Based on Eurocode 2 standard [30], concrete materials with specified strength of 17–25 MPa can be suggested for applications such as driveways, garages, and sidewalks. Among the mixes with total sand-GTR replacement, of greatest interest in terms of “greener” design and production, only RP50-RG50 mix satisfied the above requirement, demonstrating the importance in modulating the size gradation of the rubber aggregates to obtain adequate strength for technological purposes.

### 3.4. Static Mechanical Properties: Four-Point Flexural Test

**Figure 12:**
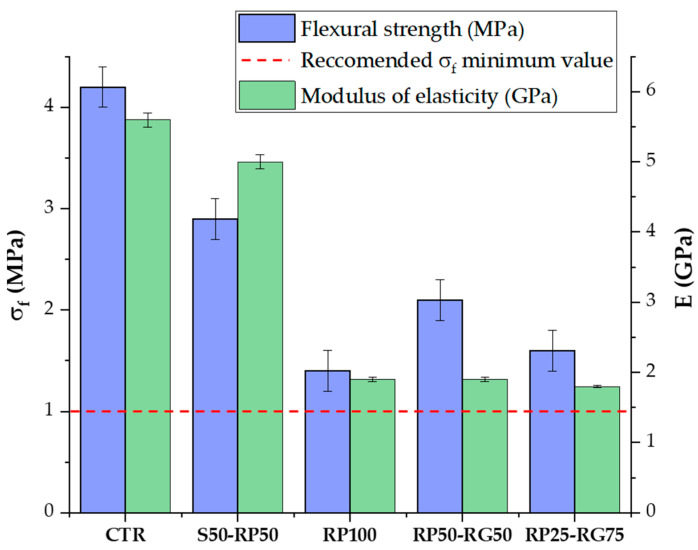
Flexural strength and modulus of elasticity: Test results. Dispersion lines in the plot represent the standard deviations.

Results of flexural strength (*σ_f_*) and modulus of elasticity (E) are shown in Figure 12. The bending strength decrease is in trend with that of *σ_c_*, which might be credited to the same reasons that influence the mechanical behavior under compression. Compared to reference sample (4.21 MPa), the increase in GTR amount implies a systematic drop in *σ_f_*. In S50-RP50 mix, the strength is 2.95 MPa with loss in strength by 30% (2.95 MPa). In RP100, RP50-RG59, and RP25-RG75 samples *σ_f_* reduces by 68% (1.37 MPa), 50% (2.11 MPa), and 63% (1.57 MPa). Like for the *σ_c_*, GTR addition maintains adequate flexural strength properties, meeting the technical criteria for pervious concrete paving applications, which require a minimum *σ_f_* of 1 MPa [28,29].

As can be seen from E experimental data, GTR-based samples obviously show lesser modulus than reference mix. This reduction can be attributed to the addition of flexible polymer particles having lower E than mineral aggregates. The sand-GTR replacement ratio would appear to have considerable control over the elastic properties of the material. The replacement of 50% volume of sand with rubber powder (S50-RP50 mix) leads to 10% reduction in E-value, compared to CTR sample. In the case of 100% *v*/*v* rubber formulations (RP100, RP50-RG50, and RP25-RG75), a “plateau” is observed in the average E-value, which ranges from 1.82 GPa (in RP25-RG75 mix) to 1.94 GPa (in RP100 mix). Hence, the rubber size gradation has an insignificant influence on the material elasticity, as also demonstrated by Ganjian et al. [53]. Low elastic stiffness may be an undesirable condition in some structural applications. For the proposals considered in this study (such as concrete members for driveway flooring), the increase in mechanical deformability can assist in a more effective way the stress distribution to the underlying foundation when the wheel load acts on the pavement blocks [54]. In addition, higher elasticity would also be an attractive feature to produce paving blocks in foot paths, jogging paths, and sport surfaces, ensuring a softer and more comfortable pavement during the activities.

### 3.5. Dynamic Mechanical Properties: Charpy Impact Test

**Figure 13:**
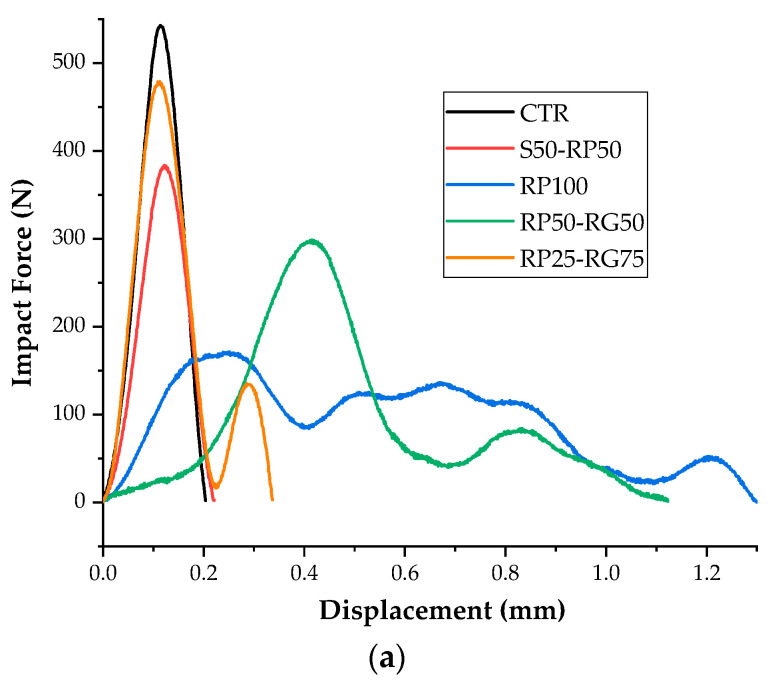

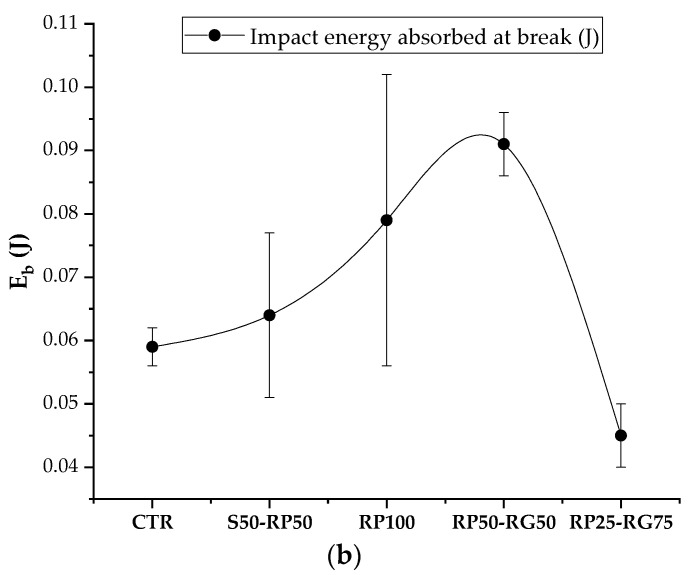
(**a**) Impact force–displacement curves and (**b**) impact energy absorbed at break: Test results.

The load–displacement graphs (Figure 13a) of GTR-cement samples obtained by Charpy impact test show two different parts: (a) a linear ascending region up to the peak force, which defines the dynamic stiffness properties of the material; and (b) a descending post-peak stroke, which highlights the deformation-at-break rate of the tested specimens. The deformability of rubberized samples is significantly larger than those of CTR mix. With respect to plain formulation, the replacement of sand by GTR gradually improves the peak strain by 7% and up to 286% for rubber volume fractions of 50% *v*/*v* (S50-RP50 mix) and 100% *v*/*v* (RP50-RG50 mix), respectively. As shown in Figure 13b, a growing trend can also be detected in terms of impact energy absorbed at break (*E_b_*). Compared to CTR mix, *E_b_* value increased by 8.5%, 34%, and 54% in S50-RP50, RP100, and RP50-RG50, respectively. The improved strain capacity and mechanical energy absorption ability result from the effect of GTR on toughness mechanisms of the cementitious material. Two plausible explanations for this evidence can be made [55,56]: (a) the elastomeric characteristic of rubber particles leads higher flexibility of rubberized mortars, considerably enhancing the energy dissipation as compared to the brittle nature of sample without GTR replacement; and (b) the polymer aggregates affect the stress field of the material under load, slowing down the kinetics of the microcracks’ propagation. Rubber particles act as obstacles to crack propagation and coalescence, improving the post-cracking deflection and energy absorption. GTR size gradation seems to be crucial on the impact performance. By comparing the totally modified mixes (0 *v*/*v*% of sand), it is observed that the ductility and *E_b_* increases from RP100 to RP50-RG50. Such result is attributable to the effect of rubber granules which, due to their larger size than rubber powder, constrain the cracks to propagate along a wider path around the particle surface, delaying the specimen failure and its brittle damage. In addition, the presence of coarse rubber accentuates the diffusion of ITZ voids which promote the stress relaxation in the matrix, enhancing the strain capacity and fracture energy dissipation [57]. However, the contribution of GTR on ductility properties would appear ineffective in the RP25-RG75 mix, where more fragile behavior and lower energy absorption than the other mixes occur. This evidence could be consistent with the following assumption: an imbalance between RP and RG induces widespread ITZ defects in the material which defunctionalizes the action of the polymer inclusions on the dynamic response. A previous study conducted by Wang et al. [58] on the interfacial adhesion mechanism between rubber aggregates and cement paste as a function of the size gradation, highlighted that increasing the size and content of coarser rubber aggregate is more remarkable in terms of incrementing the crack width in ITZ. Lack of cohesion between rubber and matrix would inhibit the load transfer between the two phases, minimizing the tough effect of the polymer particles. Further investigations can lead to reaching an optimal equilibrium between the GTR fractions that maximize the energy absorption and deformation capability without significant fail in strength.

The ability to sustain impact loads is a key requirement in the fabrication of paving elements for parking areas. Improving the toughness and the strain performance of blocks means enhancing their resistance to cracking induced by frequent wheel impacts, extending the service-life of the paving member [59]. In the design of pedestrian and sports surfaces, the energy absorbency is crucial to effectively dampen the shocks caused by the motor activities and therefore reduce the occurrence of injuries [60].

### 3.6. Acoustic Properties: Sound Absorption and Acoustic Flow Resistivity

Ordinary concrete is considered an acoustic shielding material that exhibits high sound reflection behavior, due to its high density (~2500 kg/m^3^), relatively low porosity (~9 to 10%), and low *α*-value, which typically ranges from 0.03 to 0.05 [61,62]. Although the sound insulation properties are highly attractive in many applications (rigid walls in buildings or sound barriers in highways), the attenuation of noise in urban areas and the quietness in indoor environments are mainly entrusted to materials with sound-absorbing peculiarities. As shown in previous works, the application of rubber from waste tires as natural aggregate replacement in concrete materials causes an improvement in sound absorption characteristics. Ghizdăveț et al. [46] evaluated the influence of different crumb rubber dosages (0%, 5%, and 7.5% by weight) on the acoustic absorption properties of concrete mixes. Their results highlighted the maximum value of *α*-coefficient of 0.93 (at 1500–1600 Hz) for the formulation with 5% added rubber. Wang and Du [63] found a maximum *α*-value of 0.40 (at 1000 Hz) incorporating recycled rubber with particle size of 5–10 mm size, in replacement rate of 30%. Corredor-Bedoya et al. [64] investigated the sound absorption performance of rubberized mortars containing 10%, 15%, and 25% (in mass) of rubber particles with 75–500 μm size distribution range. Mortar containing 25% of polymer fraction showed the highest values of *α* (0.20–0.40) in the medium-high frequency range.

**Figure 14:**
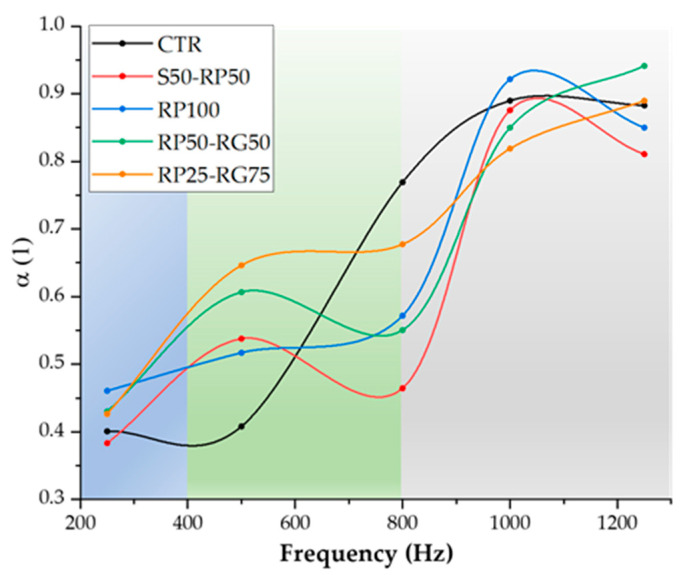
Variation of the acoustic absorption coefficient (*α*) with frequency: test results.

Sound absorption data, expressed in terms of *α* as a function of frequency, are given in Figure 14. From a global evaluation of the results, it is clear that the cementitious mixes under investigation provide *α*-coefficients from 0.38 to 0.94, classifying these materials as good sound absorbers compared to conventional concrete and confirming the literature findings previously discussed. For an exhaustive discussion of the experimental results, the *α*-spectra were analyzed in three different frequency ranges: low-frequency band (blue), medium frequency band (green), and high-frequency band (grey).

- In the low-frequency band (around 250 Hz), the lowest sound absorption performances for each class of material and irrelevant variations in terms of *α*-coefficient can be observed. The slow fluctuation of low-frequency sound waves leads to poor interaction between absorbing materials and the viscous air medium, resulting in a less effective dissipation of acoustic energy. It is known that the mitigation of low-frequency sound can be achieved by modulating the thickness of the absorbing structure [65]. Low-frequency noise results from both natural and artificial sources (such as air turbulence, thunder, earthquake, road vehicles, aircraft, industrial machinery, and air movement devices), and the adverse effects on human health are of particular concern because of its pervasiveness due to numerous sources, efficient propagation, and reduced attenuation effectiveness of many structures and components, including walls and hearing protection [66]. Considering this, obtaining sound absorption rates between 38% (S50-RP50 mix) and 46% (RP100 mix) can be considered a valuable result and a good “starting point” for optimizing materials in several civil applications for low-frequency noise control.
- At high frequencies, the acoustic absorption is mainly governed by viscous friction mechanisms between the sound wave and the pore system of the materials, which lead to dissipation of acoustic energy into thermal energy. Hence, the higher is the open porosity the higher is the sound absorption rate [62]. Over 800 Hz, all the testing formulations show the most efficient absorbing behavior (*α*-coefficients close to 1) and very similar acoustic properties, resulting from analogous *Φ*-values (see Figure 8). Then, a comparable impact in terms of microstructure-induced acoustic dissipation occurs.
- In the central range of the spectrum (around 500 Hz), an absorption peak is observed in all the mixes functionalized with GTR (*α*-coefficients of 0.54, 0.52, 0.61, and 0.65 in S50-RP50 mix, RP100 mix, RP50-RG50 mix, and RP25-RG75 mix, respectively), unlike the neat formulation which provides a flat trend (*α*-coefficient of 0.41). In this case, the rubber aggregates induced an additional mechanism in the acoustic attenuation related to their viscoelastic properties [67]. When subjected to the acoustic wave, flexible rubber particles tend to store and dissipate the vibration energy, increasing the vibro-acoustic damping in a more effective way than the stiff natural aggregates. More intense absorption peaks occur in the GTR-cement mortars functionalized with RG, indicating a more relevant effect of the coarse polymer aggregate on the acoustic behavior, in agreement with Habib et al. [68]. Enhancing the acoustic absorption properties in this spectral range can be exploited in the design of non-structural acoustic components (such as quiet brick pavements) intended for parking lots. Indeed, as reported in the study conducted by Baltrenas et al. [69], the noise levels in the parking areas (movements of vehicles maneuvering within the space, closing of doors and boots, starting or revving, and idle engines) reach the maximum values (close to 90 dB) in the frequency range from 50 Hz to 500 Hz.

Based on the NRC measurement results, reported in Table 3, all the cementitious samples fall within the class of “highly” sound-absorbing materials (NRC of 0.50–0.80), in accordance with the classification proposed by ASTM C423 standard [70]. Table 3 also reports the experimental values of *σ_a_*. *σ_a_* is another fundamental indicator of the sound absorption properties of an acoustical medium. It defines the resistance that airflow meets through a pervious structure. Too high *σ_a_*-value indicates an obstacle for the acoustic wave to penetrate the material; therefore, sound reflection phenomena are promoted. Very low *σ_a_*-value implies ineffective friction effect to dissipate sound energy significantly. Generally, sound-absorbing materials are identified by *σ_a_*-value between 10^3^ N s m^−4^ and 10^6^ N s m^−4^ [71]. As confirmation of the effective sound-absorbing characteristics of the mixes investigated in this work, it was found that all *σ_a_* indices fall within this range. By comparing the experimental values, the addition of GTR seems to slightly increase the acoustic resistivity of the material with respect to the plain formulation. Two reasons could be explained this evidence:

- The flow resistance increases with decreasing the pore size. This improvement occurs because, for an acoustic medium with small-sized pores, the frictional losses are high and a considerable part of the acoustic wave that propagates into the pore system is dissipated [72]. The inference agrees with the pore size analysis results (Table 2), where a gradual reduction in the pore dimension with the addition of the rubber aggregates was observed.
- In accordance with Su et al. [73], the addition of polymer inclusions in cement matrix would lead to an increase in material’s tortuosity, making more complex the path of the acoustic wave across the sample and maximizing the fluid–structure dissipative interactions and therefore *σ_a_*-value. However, this hypothesis should be experimentally confirmed through more detailed microstructural analysis of the samples by using X-ray microtomography techniques.

### 3.7. Statistical Analysis by One-Way ANOVA Test

Based on the experimental results, the measured values of *Φ*, *WA*, *σ_c_*, *σ_f_*, and *E_b_* were selected as more representative dependent variables to statistically evaluate the influence of GTR size expressed as RP:RG proportion ratios. Table 4 summarizes the results of statistical analysis, where df indicates degree of freedom and F-value is the ratio of between group variance to within group variance. Each F-value corresponds to a *p*-value. The larger the F-value, the smaller the *p*-value, the greater the difference in characteristics between groups. Therefore, the effect of influencing variable on the response factor results more significant.

The *p*-values for *Φ*, *WA*, *σ_c_*, and *σ_f_* fall below the significance level fixed for the statistical evaluation (*p*-value < 0.05), revealing that the GTR size induces a significant effect to material characteristics in terms of microstructure and static mechanical strength. Concerning *σ_c_*, despite ANOVA analysis providing statistical significance, by observing the plot of Figure 11, the confidence intervals between RP100, RP50-RG50, and RP25-RG75 overlap, suggesting a possible discrepancy among the experimental and statistical results. It is well established that non-overlapping confidence intervals imply statistically significant difference in size of effect between groups. Conversely, the fact that error bars overlap does not necessarily imply that the mean values are not significantly different from one another. The observation of overlapping is a practice less formal, more conservative, and less accurate than the statistical assessment based on the *p*-value. Indeed, ANOVA also refers to the overlapping rate between the variation intervals to evaluate the effective statistical difference between samples subjected to the effect of influencing factors (in this case RP:RG proportion ratio) [74]. In the light of the foregoing evidence, it seems that variability levels (in terms of standard deviations) of *σ_c_* for the three mixtures under examination, although overlapping, preserve the statistical correlation between mechanical properties and rubber size gradation. The significance is not verified for *E_b_*. Although the dynamic mechanical test showed an increase in the fracture toughness of the material with the addition of GTR, from the statistical data, the effect of RP:RG ratio is less statistically significant than what was inferred in the analysis of the results. The test method may have a relevant influence on the dispersion of experimental results. Specifically, the specimens’ dimension for impact test may not be representative of the effective ratio of the two rubber fractions in the cement matrix, implying a dynamic behavior not fully attributable to GTR size gradation.

Starting from the obtained statistical and experimental findings, a possible upgrade of the research could be based on the development of a factorial design aimed at studying the effect of both the fine:coarse ratio and different particle sizes of the fine and coarse rubber aggregates to clarify in more detail the influence of the rubber size gradation and identify optimal formulations for paving block purposes.

## 4. Conclusions

In this work, the possibility of using cement mortars functionalized with ground tire rubber for the design of paving units intended in various civil applications, including parking areas, garages, sidewalks, or sports surfaces, was evaluated through a targeted laboratory characterization. Cementitious mixes were produced and studied by incorporation of 50–100% *v*/*v* of waste tire rubber particles in two different fractions (rubber powder and rubber granules) as replacement of the mineral aggregates. The major findings of this research are as follows:

- The obtained results of the vacuum saturation method of the cement mortars with and without rubber addition predicted that the permeable porosity and water absorption were not significantly affected. The examined formulations provided porosity values from 15.9% (RP100 mix) to 21.7% (RP50-RG50 mix), which are considered optimal requirements for water drainage purposes.
- Mechanical tests showed an inevitable worsening of strength performance with the addition of ground tire rubber. However, the mix designs developed in this work provided smaller strength drops than rubber-cement formulations, reported in the literature, with similar replacement levels. Furthermore, by balancing the content of the two polymer fractions it was possible to achieve suitable mechanical properties for the reference applications proposed in this work. Indeed, RP50-RG50 sample yield a compressive strength (17.9 MPa) within the range recommended by Eurocode 2 standard for paving unit applicable in driveways, garages, or sidewalks. In terms of flexural strength, rubber incorporation preserves adequate properties in each formulation, meeting the technical criteria for pervious concrete paving applications (>1 MPa).
- Rubber aggregates induce a significant improvement in toughness and strain capacity of the material under dynamic conditions. Although the influence of the coarse aggregate provides the best performance in terms of absorbed mechanical energy (54% increase in RP50-RG50 mix over the CTR sample), its content must be carefully balanced to avoid controversial effects due to the increased occurrence of interfacial defects in the matrix (as confirmed by the scanning electron microscopy analysis). The optimized ability to absorb mechanical energy under impact loads is a valuable technological feature that can enhance the pavement block performance, extending its service life and providing effective damping against the shocks caused by the motor activities.
- Due to their pervious microstructure and lightweight properties, all cement mortars investigated show an impressive sound absorption rate (acoustic absorption coefficient = 0.38–0.94) in the frequency region of 250–1250 Hz. As also demonstrated by the noise reduction coefficient and acoustic flow resistivity data, the samples are classified as “high sound-absorbable materials” and therefore potentially suitable for noise attenuation applications in the civil sector.
- The incorporation of ground tire rubber provides an additional sound absorption mechanism (acoustic damping by a viscoelastic effect), which results in *α*-coefficient peaks around 500 Hz. This feature can be exploited for noise-control interventions in parking areas.

The “added value” of this research was to demonstrate the potential technological applicability of sand-free rubberized concrete mixes with a high level of eco-sustainability and engineering functionality. By exploiting statistical factorial design approaches, in the next step of the research activity, the influence of both the fine:coarse rubber ratio and different particle sizes of the fine and coarse polymer aggregates will be investigated in detail to identify optimum mixes for lightweight paving applications. Then, the laboratory characterization will be scaled to the first block prototypes for a more in-depth characterization of the physical, mechanical, and acoustic performances.

## Figures and Tables

**Table 1:** Mix proportions.

Components	Sample ID				
	CTR	S50-RP50	RP100	RP50-RG50	RP25-RG75
GTR in total sand (% *v*/*v*)	0% *v*/*v*	50% *v*/*v*	100% *v*/*v*	100% *v*/*v*	100% *v*/*v*
					
Cement (kg/m^3^)	800	800	800	800	800
					
Water (kg/m^3^)	300	280	260	250	230
					
Water/Cement	0.375	0.350	0.325	0.313	0.287
					
Sand (kg/m^3^)	1100	550	0	0	0
					
RP (kg/m^3^)	0	150	300	150	75
					
RG (kg/m^3^)	0	0	0	160	240
					
SF (kg/m^3^)	120	120	120	120	120
					
PES (kg/m^3^)	4	4	4	4	4
					
WRA (kg/m^3^)	8	8	8	8	8
					
CEA (kg/m^3^)	20	20	20	20	20

**Table 2:** Macro-porosity by image analysis: Pore size distribution.

	Pore Size Distribution: Cumulative Frequency (%)			
Sample	<100 μm	100–200 μm	200–300 μm	>300 μm
CTR	5	37.5	32.5	25
S50-RP50	0	60	27.5	12.5
RP100	20	57.5	15	7.5
RP50-RG50	27.5	50	15	7.5
RP25-RG75	55	30	12.5	2.5

**Table 3:** Noise reduction coefficient and acoustic flow resistivity (standard deviations in round brackets): test results.

Sample	NRC	*σ_a_* (N s m^−4^)
CTR	0.670	12,030 (1130)
S50-RP50	0.614	10,948 (1760)
RP100	0.664	13,200 (2615)
RP50-RG50	0.676	13,550 (618)
RP25-RG75	0.692	12,247 (579)

**Table 4:** ANOVA test results for the effect of RP:RG proportion on various performance parameters.

Response Factor	df	Sum of Squares	Mean Square	F-Value	*p*-Value (<0.05)	Significance
*Φ* (%)	2	66.90451	33.45225	33.39416	0.00007	Yes
						
*WA* (%)	2	9.650842	4.825421	10.01817	0.00514	Yes
						
*σ_c_* (MPa)	2	86.85019	43.42509	4.490719	0.02962	Yes
						
*σ_f_* (MPa)	2	0.921553	0.460777	10.67709	0.01055	Yes
						
*E_b_* (J)	2	0.003494	0.001747	3.117985	0.11791	No

## Data Availability

The data presented in this study are available on request from the corresponding author.
